# Cerebral Perfusion Changes After Osteopathic Manipulative Treatment: A Randomized Manual Placebo-Controlled Trial

**DOI:** 10.3389/fphys.2019.00403

**Published:** 2019-04-05

**Authors:** Federica Tamburella, Federica Piras, Fabrizio Piras, Barbara Spanò, Marco Tramontano, Tommaso Gili

**Affiliations:** ^1^IRCCS Fondazione Santa Lucia, Rome, Italy; ^2^IMT School for Advanced Studies Lucca, Lucca, Italy

**Keywords:** arterial spin labeling, osteopathic manipulative treatment, placebo, posterior cingulate cortex, somatic dysfunction

## Abstract

Osteopathic Manipulative Treatment (OMT) is a therapeutic approach aimed at enhancing the body’s self-regulation focusing on somatic dysfunctions correction. Despite evidence of OMT effectiveness, the underlying neurophysiological mechanisms, as well as blood perfusion effects, are still poorly understood. The study aim was to address OMT effects on cerebral blood flow (CBF) in asymptomatic young volunteers as measured by Magnetic Resonance Arterial Spin Labeling (ASL) method. Thirty blinded participants were randomized to OMT or placebo, and evaluated with an MRI protocol before manual intervention (T0), immediately after (T1), and 3 days later (T2). After T0 MRI, participants received 45 min of OMT, focused on correcting whole body somatic dysfunctions, or placebo manual treatment, consisting of passive touches in a protocolled order. After treatment, participants completed a de-blinding questionnaire about treatment perception. Results show significant differences due to treatment only for the OMT group (OMTg): perfusion decreased (compared to T0) in a cluster comprising the left posterior cingulate cortex (PCC) and the superior parietal lobule, while increased at T2 in the contralateral PCC. Furthermore, more than 60% of participants believed they had undergone OMT. The CBF modifications at T2 suggest that OMT produced immediate but reversible effects on CBF.

## Introduction

Osteopathic Manipulative Treatment (OMT) is a therapeutic approach aimed at enhancing body’s self-regulation aligned with the principles of practice and application of five models of the structure-function relationship (Thomson et al., 2011; Lunghi et al., 2016; Hruby et al., 2017). These models (biomechanical, respiratory/circulatory, neurological, biopsychosocial, and bioenergetic) are typically used in combination to provide a framework for interpreting the significance of a somatic dysfunction (ICD-10 Clinical Modification, 2010) guiding the osteopath through diagnosis and treatment. The OMT techniques are mainly focused on correcting the somatic dysfunctions using articular and myofascial techniques, balanced ligamentous tension and osteopathy in the cranial field. The effectiveness of OMT has already been studied on several clinical conditions such as primary headache (Cerritelli et al., 2017b; D’Ippolito et al., 2017; Tassorelli et al., 2017) and chronic low-back pain (Franke et al., 2014; Licciardone et al., 2016; Task Force on the Low Back Pain Clinical Practice Guidelines, 2016). The main advantage for patients is the effective relief of acute and chronic pain (Cerritelli et al., 2016a; Ruffini et al., 2016). Further positive effects consisted in the reduction of hospitalization length and related costs, in a large population of preterm infants (Lanaro et al., 2017) and in the management of newborns’ pain (Cerritelli et al., 2015).

Despite the evidence of OMT effectiveness, the neurophysiological mechanisms underlying clinical improvements are poorly understood. A recently published crossover study (Ponzo et al., 2018) showed that OMT intervention on volunteers with somatic dysfunction was able to enhance the corticospinal excitability produced by Transcranial Magnetic Stimulation (TMS).

However, neuroimaging evidence of OMT-induced brain changes is still scarce. A study assessing the brain cortical activity by electroencephalography after osteopathic intervention in the cranial field showed an increase in the absolute power of alpha rhythm (Miana et al., 2013), thus indicating that OMT can induce changes in oscillatory neural activity. Cerebral tissue oxygenation after OMT was assessed by Shi et al., showing a progressive reduction of oxygen saturation in prefrontal lobes, bilaterally (Shi et al., 2011). Cerebral perfusion changes have also been demonstrated in a specific touch-based trial showing significant effects on the subjects’ functional connectivity patterns in cortical areas processing the interoceptive and attentional value of touch [i.e., the insula and the posterior cingulate cortex (PCC)] (Cerritelli et al., 2017a) but no OMT effects were assessed.

Although Positron Emission Tomography (PET) represents the gold standard to investigate cerebral perfusion, Arterial Spin Labeling (ASL) involves the utilization of an endogenous tracer, thus avoiding the risks associated with exogenous radioactive ones. ASL uses magnetically labeled arterial blood water and changes in its decay as a measure of cerebral perfusion. ASL is specific to intravascular changes and can provide absolute quantification of perfusion values. Since this involves the pair-wise subtraction of control and tagged images, baseline drift and motion artifacts do not affect ASL, making it suitable for long term repetitive studies or for those with low-frequency changes (Detre and Wang, 2002). ASL based techniques are especially appropriate for studies on the physiology of neuronal activity whenever an independent, action-related effect on perfusion is expected.

For this reason, we hypothesize that using an ASL based technique we could deeper understand which are the possible neurophysiological OMT effects in pain-free subjects. Our hypothesis is that OMT could induce cerebral perfusion effects through a parasympathetic/sympathetic modulation. While the neurophysiological aspects of several types of touches were largely studied (Gay et al., 2014; McGlone et al., 2014; Lamm et al., 2015), the effect of OMT on brain perfusion has never been investigated. Therefore, in the light of the above, the aim of this study is to explore the potential changes induced by OMT in cerebral perfusion, by measuring ASL in asymptomatic young volunteers.

## Materials and Methods

### Ethics Statement

This randomized-controlled single blinded study was approved by the Fondazione Santa Lucia’s local ethic committee with protocol number CE/PROG.625 and was conducted in accordance with the Declaration of Helsinki.

### Subjects

All interventions were performed at the outpatient clinic of Fondazione Santa Lucia (Scientific Institute for Research and Health Care) from September 2017 to June 2018. Participants were recruited at the Tor Vergata University of Rome. The recruitment document explained that participation was voluntary, without incentives for participants, and dependent on the inclusion and exclusion criteria. All interested participants received information about the project by telephone and were briefly interviewed by a clinician not involved in the intervention sessions, to assess eligibility according to the inclusion and exclusion criteria (see below). Before participating, volunteers provided written informed consent. Forty-four asymptomatic, non-smoker, osteopathically naïve volunteers were recruited. No subject was under any pharmacological treatment during the previous 4 weeks, or suffered from pain within the 6 months before the enrolment.

The inclusion criteria were: age between 18 and 40 years and suitability for MRI scanning. Exclusion criteria included: (i) cognitive impairment, based on Mini Mental State Examination (MMSE) (Folstein et al., 1975) score ≤ 24 according to norms for the Italian population (Measso et al., 1993), and confirmed by a deeper clinical neuropsychological evaluation using the Mental Deterioration Battery (Carlesimo et al., 1996), and NINCDS-ADRDA criteria for dementia (McKhann et al., 2011); (ii) subjective complaints of memory difficulties, or of any other cognitive deficit, interfering or not, with daily living activities; (iii) major medical illnesses, e.g., diabetes (not stabilized), obstructive pulmonary disease, or asthma; hematologic and oncologic disorders; pernicious anemia; clinically significant and unstable active gastrointestinal, renal, hepatic, endocrine, or cardiovascular system diseases; newly treated hypothyroidism; (iv) current or reported psychiatric [assessed by the SCID-II (First et al., 1997)] or neurological (assessed by a clinical neurological evaluation) disorders (e.g., schizophrenia, mood disorders, anxiety disorders, stroke, Parkinson’s disease, seizure disorder, head injury with loss of consciousness, and any other significant mental or neurological disorder); (v) known or suspected history of alcoholism or drug dependence and abuse during lifetime; (vi) MRI evidence of focal parenchymal abnormalities or cerebro-vascular diseases: for each participant, a trained neuroradiologist and a neuropsychologist expert in neuroimaging co-inspected all the available clinical MRI sequences (i.e., T1- and T2- weighted and FLAIR images) to ensure that participants were free from structural brain pathology and vascular lesions (i.e., FLAIR or T2-weighted hyper-intensities and T1-weighted hypo-intensities). Participants were asked to avoid the use of contraceptive drugs, alcohol, nicotine, or other substance abuse during the study.

### Experimental Design

Participants were randomly divided into two groups: the OMT group (OMTg) and the placebo group (Pg). Block randomization was performed according to a computer-generated pseudo-randomized list. Participants were unaware of the study design and outcome, as well as of group allocation. A researcher not involved in the intervention sessions performed the randomization. He was the only responsible for the process and securely stored the randomization list.

All participants underwent an MRI session before the intervention (baseline or T0), immediately after (T1), and after 3 days (T2). Between T0 and T1 each participant received a single session of 45 min of OMT or placebo manual treatment.

The OMT session was performed by two female healthcare professionals who had completed a training program in osteopathy aligned with Italian Core Competencies in osteopathy (Sciomachen et al., 2018) and with European Standard on Osteopathic Healthcare Provision.

Somatic dysfunctions were addressed according to tissue alteration, asymmetry, range of motion and tenderness parameters (TART) which guided the osteopathic evaluation and intervention (Educational Council on Osteopathic Principles [ECOP], 2011). Somatic dysfunctions were detected in the whole body, then balanced one by one to define a primary order of treatment according to TART parameters. For each participant, osteopaths used the outpatient osteopathic SOAP (subjective, objective, assessment, plan) note form. OMT techniques were focused on correcting the dysfunctions found during the initial physical examination and included articular and myofascial techniques, balanced ligamentous tension, visceral manipulations, and osteopathy in the cranial field (see Supplementary Table S1) (Magoun, 1976; Lay, 1997; Johnson and Kurtz, 2003; Sciomachen et al., 2018). The manual placebo treatment was performed by the same osteopaths and consisted of a passive touch without joint mobilization in a protocolled order (Noll et al., 2004). The osteopaths were standing next to the bed, they touched lumbar and dorsal spine of the subjects in prone position for 10 min, and then, in supine position, they touched for 10 min the shoulders, the hips; then the neck, the sternum, and the chest were touched for 5 min each. A researcher specifically trained the osteopaths on the placebo protocol.

No adverse effects were reported for any participant.

### De-Blinding Questionnaire

After each treatment session, participants were asked to complete the de-blinding questionnaire administered exclusively by a treatment-blinded external trained psychologist not involved in the intervention. The questionnaire consisted of three consecutive questions about subjects’ perception of the treatment received. After being questioned on whether according to their perception, they thought they have received “OMT” or “Placebo” treatment, subjects were asked on a 0–10 numeric rating scale (NRS), where 0 represented absolutely uncertainty and 10 represented absolutely certainty (Hrobjartsson, 2002; Chaibi et al., 2015), how certain they were regarding group allocation. Finally, they were asked to rate the perceived usefulness of the treatment received, based on a 0–10 NRS, where 0 represented absolutely useless and 10 represented absolutely useful.

### MRI Data Collection

Data were recorded using a Philips Achieva 3T MRI scanner using a 32 channels receive-only head coil. Whole brain cerebral blood flow (CBF) was measured using pseudo-continuous arterial spin (pcASL). Sixty- tag-control image pairs with 29 axial slices (3 × 3 × 4.5 mm voxel resolution, matrix 80 × 80, with a 0.5 mm inter-slice gap) were acquired (TR/TE = 4000/10 ms, label duration = 1650 ms, and post-label delay = 1525 ms). A separate single shot EPI (M0) scan was acquired (TR = ∞) with the same parameters to measure the equilibrium brain tissue magnetisation for calibration purposes. A high-resolution T1-weighted whole-brain structural image was also recorded (1 × 1 × 1 mm voxels). In-house software was used to calculate the mean difference of the motion-corrected (MCFLIRT (Jenkinson et al., 2002)) tag-control perfusion pair time-series. Conversion to CBF in ml/100 g/min was done by means of the Oxford_asl part of the BASIL toolbox (Chappell et al., 2009) within FSL^1^. Perfusion data were transformed, first, from ASL space to individual subjects’ structural space using FLIRT (FMRIB’s Linear Registration Tool) and then non-linearly to a standard space (Montreal Neurological Institute MNI152 standard map) using FNIRT (FMRIB’s Non-Linear Registration Tool).

### Power Analysis

To determine a sufficient sample size for two sample *t-tests*, power analysis was conducted using G^∗^Power (Faul et al., 2007) based on a previous paper by Shi et al. (2011), where cranial osteopathic manipulative suppression techniques determined a significant decrease in cerebral tissue oxygen saturation in both prefrontal lobes with a large effect size (Cohen’s *d* = -0.5). Using alpha equal to 0.05, power equal to 0.80 and Cohen’s d equal to 0.55 (the OMT in our study was not restricted to osteopathy in the cranial field) the desired sample size for the difference between 2 dependent means resulted to be 22 subjects in total.

### Statistical Analysis

The statistical analyses on demographic and clinical data were performed using SPSS 21.0 for Windows (SPSS Inc., United States). Chi-square test and two-sample *t-tests* (two-tailed) were used to compare between-group differences for gender and for age and education, respectively.

Voxel-wise statistical analyses of MRI data were carried out using SPM12 (Statistical Parametric Mapping software ^2^).

Following registration of the CBF maps to the common standard space of the Montreal Neurological Institute (MNI), whole brain voxel-wise two-sample *t-tests* were performed. The interaction between the effect of “dosing” the treatment (T0, T1, T2), and the effect of “administering” the treatment (P or OMT) was modeled. The interactions are described by the contrast (OMT_T1 – OMT_T0) – (P_T1 – P_T0) for the comparison post vs. pre, (OMT_T2 – OMT _T1) – (P_T2 – P_T1) for the comparison follow up vs. post, (OMT_T2 – OMT_T0) – (P_T2 – P_T0) for the comparison follow-up vs. pre, representing treatment’s effects controlled by baseline scans, and delayed effect, respectively. We also calculated the correlation between the number of dysfunction and cerebral perfusion changes both for the contrast (OMT_T1 – OMT_T0) – (P_T1 – P_T0) and for (OMT_T2 – OMT_T1) – (P_T2 – P_T1). Data of the de-blinding questionnaire were calculated as percentages like the dichotomous “OMT” and “Placebo” data; the continuous 0–10 NRS outcomes were calculated as means for each treatment group (i.e., OMTg and Pg).

## Results

Forty-four participants were screened for eligibility. According to the inclusion/exclusion criteria, 30 participants were enrolled and randomized to OMTg and Pg groups (*N* = 15 participants in each group). No significant differences (*p* > 0.05) were found between groups in terms of age, sex, and education. Participants’ demographic characteristics are reported in Table 1.

**Table 1:** Participants’ demographic characteristics.

	OMTg (*n* = 15)	Pg (*n* = 15)	t, χ^2^
Age (years)^a^	28.0 ± 5.5	25.4 ± 3.2	^c^t_(28)_ = -1.6, *P* = 0.1 95% CI = -6.0, 0.8
Gender (M/F)^b^	8/7	4/11	^d^χ_(1)_ = 2.2, *P* = 0.3
Education (years)^a^	16.0 ± 1.5	16.1 ± 0.4	^c^t_(28)_ = 0.2, *P* = 0.9 95% CI = -0.8, 0.9
Body Mass Index	20.9 ± 6.1	21.0 ± 6.6	^c^t_(28)_ = 0.8, *P* = 0.9 95% CI = -4.6, 4.9


*Value are expressed as ^*a*^mean ± standard deviation or ^*b*^number. Group comparisons were performed with ^*c*^independent-sample *t-tests*, and ^*d*^Chi-square tests*.

**FIGURE 1:**
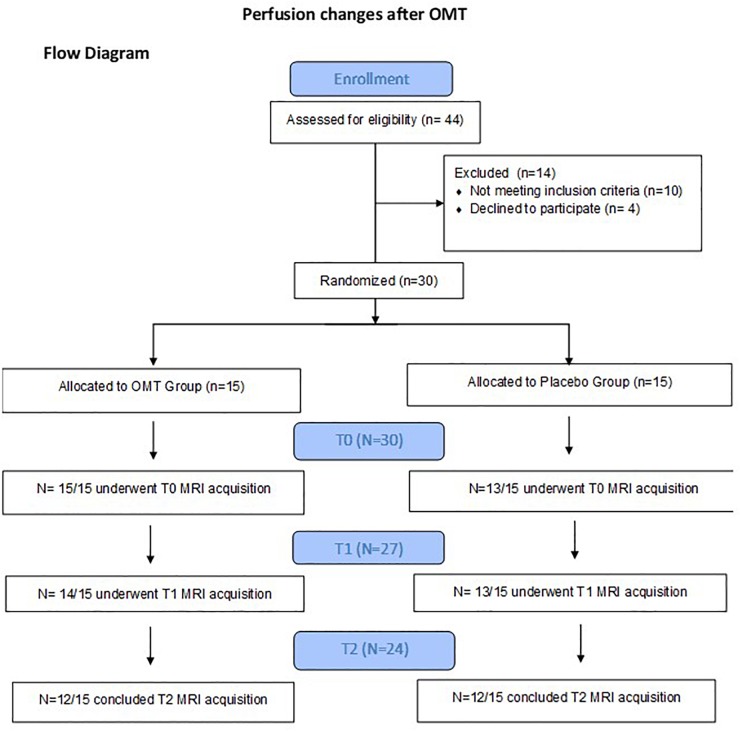
Flow Chart.

**FIGURE 2:**
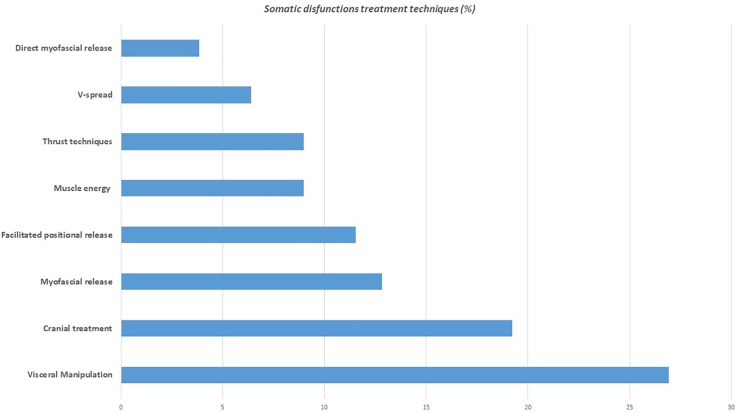
Techniques used for the treatment of the different somatic dysfunctions in OMTg (%).

Enrolment details are reported in the flow chart of the study (Figure 1). Two participants from the Pg group were excluded because referred pain perception during T0 MRI acquisition, while one participant from the OMTg dropped out at T1 due to a later developed MRI intolerance. At T2 two OMTg participants dropped out for a sudden MRI intolerance, and one Pg participant dropped out for personal reasons. For OMTg, details of the treated dysfunction with a report of the different techniques used are described in Table 2 and Figure 2, respectively.

**Table 2:** Dysfunctions localization reported for the different body segments in OMTg according to somatic dysfunction classifications.

Dysfunctions localization	%
M99.01 Cervical	28,1
M99.0 Head	26,9
M99.09 Abdomen and other regions	22,4
M99.05 Pelvic	6,4
M99.02 Thoracic	6,2
M99.03 Lumbar	3,6
M99.08 Rib cage	2,5
M99.04 Sacral	1,3
M99.06 Lower extremity	1,3
M99.07 upper extremity	1,3


**FIGURE 3:**
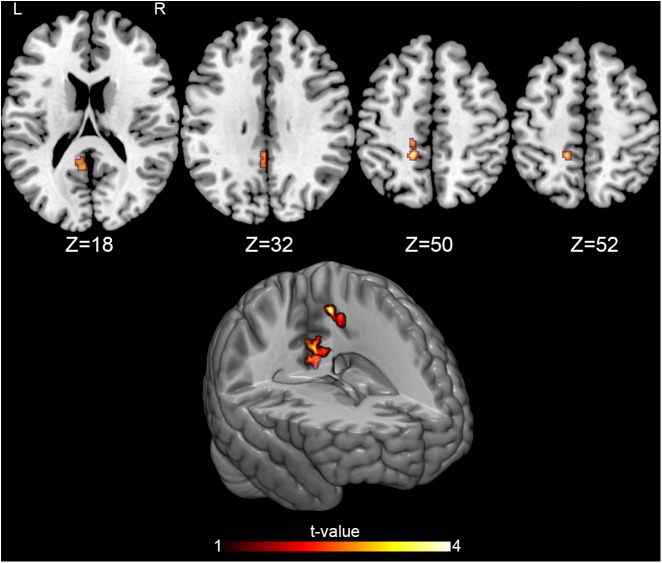
Perfusion changes induced by treatment. The figure shows the baseline-controlled group [(OMT_T1 – OMT_T0) – (P_T1 – P_T0)] differences between treatment and placebo administration, indicating a significant decrease of the CBF after treatment in two regions: the left posterior cingulate cortex (PCC) [(-4, -46, 31) MNI space coordinates] and the left superior parietal lobule [(-14, 42, 52) MNI space coordinates]. Signal changes were deemed significant at *p* < 0.005 voxel level uncorrected, corresponding to a minimum cluster size of 40.

**FIGURE 4:**
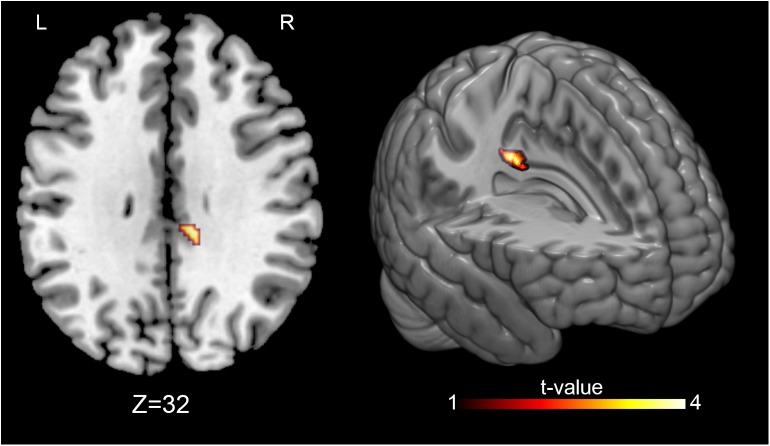
Perfusion changes induced by treatment at follow-up. The figure shows the post-treatment-follow up [(OMT_T2 – OMT _T1) – (P_T2 – P_T1)] differences between treatment and placebo administration, indicating a significant increase of the CBF at follow-up in the right PCC [(8, –30, 31) MNI space]. Signal changes were deemed significant at *p* < 0.005 voxel level uncorrected, corresponding to a minimum cluster size of 40.

MRI statistical analyses were conducted on 27 participants (14 OMTg and 13 Pg) in the comparison between T0 and T1, and on 24 participants (12 OMTg and 12 Pg) in the comparison between T1 and T2. Pre- and post-intervention changes in perfusion maps were considered as statistically significant at *p-values* of *p* < 0.005, uncorrected at the voxel level, corresponding to a minimum cluster size of 40 voxels. The (OMT_T1 – OMT_T0) – (P_T1 – P_T0) contrast revealed that perfusion decreased in a cluster of 67 voxels within the left PCC [(*x* = -4, *y* = -46, *z* = 31) MNI space coordinates] and in a cluster of 47 voxels within the left superior parietal lobule (SPL) [(*x* = -14, *y* = 42, *z* = 52) MNI space coordinates] (Figure 3). Conversely, the (OMT_T2 – OMT _T1) – (P_T2 – P_T1) contrast returned an increase of perfusion in a cluster of 45 voxels within the right PCC [(*x* = 8, *y* = -30, *z* = 31) MNI space] (Figure 4). According to our analysis, no decreases in blood perfusion were observed in T2. We also analyzed cerebral perfusion by comparing T2 vs. T0. No difference was found in the contrast (OMT_T2 – OMT_T0) – (P_T2 – P_T0), suggesting that the increase of perfusion was part of a compensatory dynamic toward the baseline condition.

Furthermore, concerning the correlation between the number of dysfunction and cerebral perfusion changes both for the contrast (OMT_T1 – OMT_T0) – (P_T1 – P_T0) and for (OMT_T2 – OMT_T1) – (P_T2 – P_T1), no significant correlation emerged.

Regarding the de-blinding questionnaire, 75% of the OMTg and 41,6% of the Pg answered that they underwent OMT. Ratings mean of both groups regarding treatment allocation certainty was 7.0 ± 1.6 and ratings means of treatment usefulness was 7.5 ± 2.6 for OMTg and 6.6 ± 1.9 for Pg.

## Discussion

This randomized controlled neuroimaging trial evaluated, for the first time, the immediate and short-term effects of OMT on cerebral perfusion. We found that OMT, but not placebo treatment, induced changes in resting cerebral perfusion measured by ASL in asymptomatic young volunteers.

In spite of the accumulating evidence suggesting OMT to be responsible for changes in the central nervous system functioning, including reflex excitability, cortical processing, as well as for changes in brain plasticity and functional connectivity (Haavik-Taylor and Murphy, 2007; Haavik and Murphy, 2011; Daligadu et al., 2013; Sparks et al., 2013; Gay et al., 2014; Niazi et al., 2015), the relationship between OMT and CBF has been scarcely investigated. Shi et al. (2011) demonstrated in healthy young adults, that specific cranial OMT with suppression or CV-4 techniques can effectively and progressively elicit the cerebral hemodynamic response by decreasing cerebral tissue oxygenation in the left and right prefrontal cortex during treatment. Moreover, cranial OMT decreased the cardiac sympathetic influence, and enhanced parasympathetic modulation, as reflected by power spectral analysis of variability in the interval between heartbeats (R-R), thus suggesting that cranial OMT could be effectively applied to modify cerebral tissue oxygen saturation and the cardiac autonomic function in healthy adults (Shi et al., 2011). Moreover, OMT intervention, as evidenced by analyzing the Heart Rate Variability, can influence the autonomic nervous system activity by increasing parasympathetic functioning and decreasing sympathetic modulation (Ruffini et al., 2015).

In line with our hypothesis assuming a larger OMT effect, as treatment in this study was not restricted just to the cranial field, we found similar results on resting cerebral perfusion, thus reinforcing the notion that whole-body OMT interventions can locally modify the vascular activity (Ponzo et al., 2018). Our intervention was aimed at treating somatic dysfunctions using different osteopathic techniques (Educational Council on Osteopathic Principles [ECOP], 2011) in different regions of the body (see Table 2 and Figure 2) and at determining their effect on CBF. We obtained a perfusion decrease only in OMTg within the left PCC and the left SPL immediately after OMT intervention (Figure 3), and an enhancement of perfusion in the right PCC after 3 days as short-term effect (Figure 4).

Furthermore, the present results support the theoretical basis of the interrelationship between neurologic and biomechanical osteopathic structure-function models (Lay, 1997) since they show that the biomechanical aspect of OMT treatment induces neurophysiologic effects which presumably determine the clinically significant positive effects.

The theoretical functional model underlying OMT assumes a dynamic balance between the parasympathetic and sympathetic nervous systems, while hypothesizes a potentially disruptive influence of biomechanical strain on both systems, and on the dynamic equilibrium between them (Donnerer, 1992). Biomechanical stresses or imbalances may indeed affect the dynamic functioning of the body, increase energy expenditure during activity, alter proprioception and change joint structures (Norre, 1995; Rimmer et al., 1995; Degenhardt and Kuchera, 1996). OMT intervention on altered somatic system functions (body framework) could stimulate the sympathetic activity, thus determining a cascade of biological and neurological events that modulate autonomic nervous system mechanisms (D’Alessandro et al., 2016).

In our study, a decrease of the resting cerebral perfusion was found immediately after OMT intervention in a cluster comprising the PCC and the SPL, while PCC perfusion increased significantly after 3 days post-OMT. Crucially for the present evidence, the PCC is a critical node of the central autonomic network [CAN; (Benarroch, 1993)] that controls preganglionic sympathetic and parasympathetic motoneurons, being particularly involved in parasympathetic functioning (Benarroch, 1993; Beissner et al., 2013; Kumral et al., 2018). Furthermore, CAN supports visceromotor and neuroendocrine responses critical for goal-directed behavior, adaptability, and health (Benarroch, 1993; Hagemann et al., 2003).

Given the PCC key role in the CAN context, we speculate that the observed change in its perfusion might index a sympathovagal modulation with a shift toward a relatively larger sympathetic (or vagal) predominance, as a consequence of the OMT effect in overcoming the sympathetic tone. These results are apparently in contrast with previous studies that reported an increase in vagally mediated hearth rate variability (HRV) indexes during or immediately after OMT. These differences could be attributed to different factors. First, the effects on vagal activity could depend on the osteopathic techinique used (Henley et al., 2008). Previous studies are generally focused on a single type of technique, while we used different techniques for each OMT. Cervical myofascial release in healthy adults usually determines significant shifts in the sympathovagal balance from the sympathetic to the parasympathetic nervous system (Henley et al., 2008), also the upper cervical spine manipulation moderately enhances parasympathetic control of hearth rate suggesting a more predominant vagal control (Giles et al., 2013). Besides, OMT induces a faster recovery of heart rate and increases sympathovagal balance after an acute mental stressor (Fornari et al., 2017). Conversely, high-velocity low-amplitude (HVLA) cervical manipulations have been shown to shift sympathovagal balance toward a more sympathetic predominance (Budgell and Hirano, 2001; Budgell and Polus, 2006). Furthermore, besides HRV, the relationship between OMT and autonomic control has been previously addressed using other different approaches. For example, a single study specifically analyzed the effect of HVLA techniques administered at the dorsal spinal level on pupil diameters, founding no OMT effects on the sympathetic nervous system in subjects with chronic neck pain. Systemic arterial blood oxygen saturation was the outcome measure in another single study (Shi et al., 2011) demonstrating that cranial suppression technique is effective in progressively reducing cerebral tissue oxygenation.

In our study, we used ASL technique, and the mechanisms behind the relationship between HRV and cerebral blood perfusion are still an open question (Allen et al., 2015). Consequently, it is not easy to frame our results in the light of previous studies. Allen et al. (2015) evidenced that resting state cerebral perfusion in many brain regions, such as the cingulate and the medial prefrontal cortex, positively correlated with vagal reactivity during tasks involving sensory/motor processing, and was negatively correlated with resting vagal function. Our hypothesis is that the observed decrease in PCC perfusion immediately after OMT intervention might indicate a vasoconstriction effect consequent to the initial sympathetic response. The increased perfusion in the same area 3 days after intervention may be indicative of a diminished sympathetic adrenergic inhibition, and less sympathetic adrenergic vasoconstriction of cerebral arteries, resulting in higher resting cerebral perfusion (Allen et al., 2015). The CBF modifications at T2 suggest that the increase of perfusion was part of a compensatory dynamic toward the baseline condition demonstrating a reversible effect of OMT on CBF.

Indeed, while the cingulate cortex is a pivotal region for emotion recognition and pain perception (Vogt, 2005), the PCC is considered an emotional pre-processor to assess self-relevance of emotional events and stimuli, as its functional inactivation could be one of the mechanisms for reducing the overall perception of noxious stimulation (Vogt, 2005). Even if the exact mechanisms underlying the OMT effect in our study are largely hypothetical, we can also speculate that the observed CBF changes in PCC are closely related to the somatic dysfunctions treatment. As the presence of somatic dysfunctions in asymptomatic individuals has biomechanical and neurological consequences, such as changes in tissue texture and activation of nociceptors promoting tissue inflammation (Fryer, 2016), we might hypothesize that the treatment of somatic dysfunctions may be responsible for central plastic changes (Haavik Taylor and Murphy, 2007; Taylor and Murphy, 2010a,b), thus producing the reported CBF modification in the PCC.

Perfusion was reduced at T1 assessment also in the SPL, an area primarily involved in visuomotor functions and spatial cognition, and specifically implicated in processing the spatial configuration of the body (Wang et al., 2015). Specifically, the SPL is related to the generation and maintenance of the body image (Wolpert et al., 1995) resulting from the integration of visual and proprioceptive inputs as to dynamically update the body image represented in SPL (Shimada et al., 2005). Such self-body image facilitates a sense of ownership [i.e., the feeling that an image of our body is, in fact, our own (Gallagher, 2000)], and a recent study suggested that the PCC is also involved in the sense of body ownership (Vogt, 2005), thus establishing a functional relationship between these two regions. According to our results, we can only hypothesize that OMT somato-sensory afferences lead to transitory effects on inputs integration and body ownership processes reflected by similar perfusion effects on both SPL and PCC areas. It is possible that an initial re-organization due to OMT generates immediate effects on the self-body image and on the sense of body ownership, as well as sympathetic effects, even if the short-term effects observed in cerebral perfusion of PCC and SPL were different. Probably, OMT specific effects on the SPL are transitory, suggesting that OMT influences the body spatial mental representation only transiently, given the observed relapsing to initial CBF level in the SPL 3 days after treatment. Conversely, the short-term effects on the PCC perfusion might underline a longer influence on CAN as increased perfusion in this region was delayed in time.

The results of the de-blinding questionnaire could support the hypothesis of a specific bottom-up OMT effect respect to the manual-placebo intervention. The 42% of participants of Pg group believed they underwent OMT, and both groups were sufficiently sure of the usefulness of the treatment received. According to a previous study (Noll et al., 2004), the placebo treatment adopted was a whole-body manual intervention, had the same duration of OMT, and the osteopaths were specifically trained to perform manual placebo intervention. However, guidelines to design the most reliable placebo for manual clinical trials are needed to increase the internal validity, and improve the external validity of findings (Cerritelli et al., 2016b) of placebo-controlled studies.

In conclusion, in asymptomatic young volunteers OMT generates a significant effect on PCC perfusion, starting from a CBF reduction immediately after manipulation, followed by an increase of CBF as short-term effect. This opposite effects may be related to a parasympathetic/sympathetic modulation induced by OMT.

Even if we provide evidence to support OMT effects on CBF, the inclusion of asymptomatic young volunteer participants may limit the implications of the present study findings regarding the relationship between OMT and brain perfusion. More obvious responses to general OMT intervention might be elicited in participants with pain, and further studies should investigate whether these changes correlate with beneficial clinical outcomes, thus being interpretable as a consequence of somatic dysfunctions correction, and ascribable to the normalization of aberrant afferent input to the CNS. However, the present is the first study in which a follow-up examination was performed 3 days after OMT, allowing the direct observation of short-term treatment effects on CBF in asymptomatic subjects, and thus favoring the notion that the osteopathic treatment has the potential to determine lasting effects beyond that of manipulation itself. Indeed, the fact that perfusion changes were observed in a cortical area involved in the dynamic balance between the sympathetic and parasympathetic systems would suggest an OMT corrective effect on the disruptive influence that biomechanical strains have on those systems. Forthcoming studies will address the potential OMT effects on both HRV and CBF, as to more thoroughly investigate manipulations consequences.

Furthermore, it would be interesting to investigate the effect of personal beliefs on CBF and to submit de-blinding questionnaires also immediately after OMT or placebo treatment, given the opposite OMT treatment responses at T1 and T2 in terms of cerebral perfusion. As a matter of fact, since blinding perception may change during the study, it would be important to ascertain whether changes in perfusion in areas involved in internally directed attention (like the SPL) might be ascribable to changes in attributed self-relevance and efficacy of the received treatment. Future studies are needed to clarify these aspects.

## Ethics Statement

This randomized-controlled single blinded study was approved by the Fondazione Santa Lucia’s local ethic committee with protocol number CE/PROG.625 and was conducted in accordance with the Declaration of Helsinki.

## Author Contributions

FT and MT organized the database. FT, FaP, and TG performed the statistical analysis. MT wrote the first draft of the manuscript. FT, FeP, FaP, BS, and TG wrote sections of the manuscript. All authors contributed to manuscript revision, read, and approved the submitted version. All authors contributed to the conception and design of the study.

## Conflict of Interest Statement

The authors declare that the research was conducted in the absence of any commercial or financial relationships that could be construed as a potential conflict of interest.

## Footnotes

1 www.fmrib.ox.ac.uk/fsl

2 www.fil.ion.ucl.ac.uk/spm/
