# Supplementary material for: Cerebral Perfusion Changes After Osteopathic Manipulative Treatment: A Randomized Manual Placebo-Controlled Trial
Source: Front Physiol. 2019 Apr 5;10:403. doi: 10.3389/fphys.2019.00403 (PMC6460882; doi:10.3389/fphys.2019.00403)
Supplement: Supplementary file 1 [file Table_1.docx]

SUPPLEMENTARY MATERIAL

*Table for Supplementary Material: Anatomical regions treated by practitioners according to the International statistical classification of diseases for the 15 participants who underwent OMT, including 3 subjects who did not complete whole MRI assessments.*

|  | Anatomical regions according to the International statistical classification of diseases | | | | | | | | | |
| --- | --- | --- | --- | --- | --- | --- | --- | --- | --- | --- |
| Participants | M99.0 Head | M99.01 Cervical | M99.02 Thoracic | M99.03 Lumbar | M99.04 Sacral | M99.05 Pelvic | M99.06  Lower extremity | M99.07  Upper extremity | M99.08 Rib cage | M99.09  Abdomen and other regions |
| OMT_1 |  | X | X | X |  | X |  |  |  | X |
| OMT_2 |  | X | X | X |  |  |  |  |  | X |
| OMT_3 |  | X |  |  |  |  |  |  |  | X |
| OMT_4 |  | X |  |  |  |  |  |  |  | X |
| OMT_5 |  | X | X |  |  |  |  | X |  | X |
| OMT_6 |  | X |  |  |  |  |  |  |  | X |
| DROP_1 | X | X |  |  | X | X |  |  |  | X |
| OMT_7 | X | X |  |  |  |  |  |  |  | X |
| OMT_8 | X |  |  |  |  |  |  |  |  | X |
| OMT_9 |  | X |  |  |  |  |  |  | X | X |
| OMT_10 | X |  | X | X |  |  |  |  |  | X |
| OMT_11 | X |  |  |  |  | X |  |  |  |  |
| OMT_12 | X |  |  |  |  |  | X |  |  | X |
| DROP_2 | X |  | X |  |  |  |  |  |  | X |
| DROP_3 | X |  |  |  |  |  |  |  |  | X |

*Table for Supplementary Material: Osteopathic Manipulative Techniques used for the treatment of the somatic dysfunctions*

| Osteopathic techniques used | | | | | | | | |
| --- | --- | --- | --- | --- | --- | --- | --- | --- |
| Participants | Direct myofascial release | V-spread | Thrust techniques | Muscle Energy | Facilitated Positional Release | Myofascial release | Cranial Treatment | Visceral Manipulation |
| OMT_1 |  |  |  | X |  | X |  | X |
| OMT_2 |  |  | X | X | X | X | X | X |
| OMT_3 |  |  |  |  |  | X |  | X |
| OMT_4 | X |  |  |  |  |  |  | X |
| OMT_5 |  |  |  |  | X | X |  | X |
| OMT_6 | X |  |  |  |  | X |  | X |
| DROP_1 |  |  |  | X |  |  |  | X |
| OMT_7 |  |  | X | X |  |  | X | X |
| OMT_8 |  |  |  |  |  | X | X | X |
| OMT_9 | X |  |  |  | X |  |  | X |
| OMT_10 |  | X | X |  |  |  | X | X |
| OMT_11 |  | X |  |  |  |  | X |  |
| OMT_12 |  |  |  |  | X |  | X | X |
| DROP_2 |  | X | X |  |  |  | X | X |
| DROP_3 |  |  |  |  | X |  | X | X |

*Table for Supplementary Material: Definition of Osteopathic Manipulative Treatment techniques*

| Definition of Osteopathic Manipulative Treatment techniques | |
| --- | --- |
| Direct myofascial release | A system of diagnosis and treatment which engages continual palpatory feedback to achieve release of myofascial tissues. |
| V-spread | Technique using forces transmitted across the diameter of the skull to accomplish sutural gapping |
| Thrust techniques | An osteopathic technique employing a rapid, therapeutic force of brief duration that travels a short distance within the anatomic range of motion of a joint, and that engages the restrictive barrier in one or more planes of motion to elicit the release of restriction. Also known as thrust technique. |
| Muscle Energy | A form of osteopathic manipulative diagnosis and treatment in which the patient’s muscles are actively used on request, from a precisely controlled position, in a specific direction, and against a distinctly executed physician counterforce |
| Facilitated Positional Release | A system of indirect myofascial release treatment. The component region of the body is placed into a neutral position, diminishing tissue and joint tension in all planes, and an activating force (compression or torsion) is added. |
| Myofascial release | A system of diagnosis and treatment first described by Andrew Taylor Still and his early students, which engages continual palpatory feedback to achieve the release of myofascial tissues. |
| Cranial Treatment | A system of diagnosis and treatment by an osteopathic practitioner using the primary respiratory mechanism and balanced membranous tension |
| Visceral Manipulation | A system of diagnosis and treatment directed to the viscera to improve physiologic function. Typically, the viscera are moved toward their fascial attachments to a point of fascial balance |
